# Draft Genome Sequence of the *Bactrocera oleae* Symbiont “*Candidatus* Erwinia dacicola”

**DOI:** 10.1128/genomeA.00896-16

**Published:** 2016-09-15

**Authors:** Frances Blow, Anastasia Gioti, David Starns, Michael Ben-Yosef, Zohar Pasternak, Edouard Jurkevitch, John Vontas, Alistair C. Darby

**Affiliations:** a Institute of Integrative Biology, University of Liverpool, Liverpool, Merseyside, United Kingdom; b Institute of Molecular Biology & Biotechnology, Foundation for Research & Technology Hellas, Heraklion Crete, Greece; c Department of Entomology, The Hebrew University of Jerusalem, Rehovot, Israel; d Department of Plant Pathology and Microbiology, Robert H. Smith Faculty of Agriculture, Food and Environment, The Hebrew University of Jerusalem, Rehovot, Israel; e Department of Crop Science, Agricultural University of Athens, Athens, Greece

## Abstract

“*Candidatus* Erwinia dacicola” is a *Gammaproteobacterium* that forms a symbiotic association with the agricultural pest *Bactrocera oleae*. Here, we present a 2.1-Mb draft hybrid genome assembly for “*Ca.* Erwinia dacicola” generated from single-cell and metagenomic data.

## GENOME ANNOUNCEMENT

The association between *Bactrocera oleae* (*Tephritidae*) and a bacterial symbiont was first discovered in 1909 (1). Several studies have since identified this organism as “*Candidatus* Erwinia dacicola” (*Enterobacteriaceae*) (2–4). “*Ca.* Erwinia dacicola” plays a role in nutrient provisioning, particularly during juvenile development in unripe olives, where it is essential for larval survival (5–8). However, due to a lack of genomic resources and the inability to culture “*Ca.* Erwinia dacicola,” the metabolic basis of its association with *B. oleae* remains elusive. We present a draft of the “*Ca.* Erwinia dacicola” genome sequence that will inform future investigations into the functional and evolutionary foundations of the symbiosis.

Multiple single-cell (eight) and metagenomic (two) libraries were used to generate a draft hybrid genome assembly of “*Ca.* Erwinia dacicola.” Single-cell libraries were prepared from the guts of adult female flies collected in Heraklion, Greece, stained with CellTracker deep red, and sorted on a Sony SH800. Genomic DNA was amplified using the REPLI-g kit (Qiagen) and was validated as “*Ca.* Erwinia dacicola” by amplification of the 16S rRNA gene, followed by digestion with the restriction enzyme PstI (9). Shotgun libraries were then prepared with the NEBNext Ultra DNA kit (New England Biosciences) and sequenced on an Illumina MiSeq sequencer at the Centre for Genomic Research, University of Liverpool, United Kingdom. Metagenomic shotgun and mate-pair (2- to 6-kb) libraries were prepared from gastric ceca dissected from third-instar larvae isolated in Israel from unripe olives and from a mixture of ripe and unripe olives, respectively. DNA for the shotgun library was extracted using an adapted cetyltrimethylammonium bromide (CTAB) method (10) with additional bead beating and lysozyme digestion, and the library was prepared with the Ovation rapid DR multiplex system (NuGen). The mate-pair library was prepared with the gel-free NexteraMate protocol from DNA extracted with the Chemagic DNA bacteria kit (Chemagen). Both libraries were prepared and sequenced on an Illumina MiSeq sequencer by LGC Genomics GmbH (Berlin, Germany).

Reads were assembled with SPAdes version 3.7.1 (11) in single-cell mode. Using Blobology (12), contigs identified as belonging to other organisms based on coverage and G+C content were excluded, and the 12,519,932 reads that mapped back to putative “*Ca.* Erwinia dacicola” contigs were extracted and reassembled with SPAdes. The result was a 2.1-Mb assembly comprising 333 scaffolds (>500 bp) at ~1,000× coverage, with an *N*_50_ of 9,998 bp. The assembly was assessed as in reference 13 and was found to be 92% complete in comparison to free-living bacteria and 100% complete in comparison to the endosymbiotic bacteria of aphids and tsetse flies, *Buchnera aphidicola* and *Wigglesworthia glossinidia*, respectively. Its G+C content (53.5%) is similar to that observed in other members of the *Erwinia* genus (14) and higher than that of other vertically transmitted endosymbiotic bacteria (15). The genome contains 2,407 protein-coding genes and 28 RNA-coding genes, based on annotation with PROKKA version 1.5.2 (16).

### Accession number(s).

This whole-genome shotgun project has been deposited at DDBJ/ENA/GenBank under the accession no. MAZB00000000. The version described in this paper is version MAZB01000000 and BioProject no. PRJNA326914.
